# Genetic evidence for behavioral preference factors on fatigue: A Mendelian randomization study of chronotype, morning alertness, and outdoor exposure

**DOI:** 10.1097/MD.0000000000049897

**Published:** 2026-08-07

**Authors:** Tao Luo, Fen Zhang, Hui Xue, Lanqing Wang

**Affiliations:** a Department of Orthopedics, Zhongnan Hospital of Wuhan University, Wuhan, 430071, China; b Department of Pathology, Zhongnan Hospital of Wuhan University, Wuhan, 430071, China; c Zhongnan Hospital of Wuhan University, Wuhan, 430071, China.

**Keywords:** behavioral preference factors, chronotype, fatigue, Mendelian randomization, outdoor exposure

## Abstract

Fatigue is a multifactorial condition influenced by environmental, behavioral, genetic, and disease-related factors. While observational studies have identified key contributors like circadian disruption, sleep disturbances, and genetic predisposition, the causal relationships remain unclear. Mendelian randomization (MR) offers a robust approach to overcome limitations of traditional studies and establish causal links between modifiable behavioral preferences and fatigue. This study employed univariate and multivariate MR (MVMR) analyses using publicly available genome-wide association study (GWAS) summary statistics to investigate the potential causal relationships between these behavioral preference factors and the risk of fatigue. We obtained GWAS summary statistics for relevant variables from the Neale Lab and MRC Integrated Epidemiology Unit (MRC-IEU) databases. After systematic screening of multiple domains (internal microenvironment, indoor environment parameters, and natural environment characteristics), the inverse variance weighted (IVW) method served as the primary analysis, complemented by sensitivity analysis (heterogeneity test, pleiotropy analysis, leave-one-out analysis, and MR-PRESSO) to evaluate result robustness. Using GWAS data from 32 traits (1645,048 participants), we identified three significant behavioral preference-related determinants of fatigue: chronotype, ease of getting up in the morning, and time spent outdoors in summer. The MR results demonstrated: Protective effects against fatigue associated with greater ease of getting up in the morning (OR = 0.991, 95%CI 0.987–0.995; *P* < .001) and longer summer outdoor exposure (OR = 0.996, 95%CI 0.992–1.000; *P* = .030); Elevated fatigue risk,linked to evening chronotype (OR = 1.003, 95%CI 1.001–1.005; *P* = .013). MVMR analysis showed that after jointly incorporating variables, the impact of ease of getting up in the morning on fatigue remained significant (OR = 0.987, 95%CI: 0.980–0.995, *P* = .002). Sensitivity analyses confirmed the robustness of these findings: although significant heterogeneity was detected for ease of getting up in the morning (Cochran’s Q test *P* < .05), no evidence of horizontal pleiotropy (MR-Egger intercept *P* > .05) or outlier SNPs (MR-PRESSO) was found, and results were consistent across multiple MR methods. These findings provide genetic evidence supporting causal relationships between modifiable behavioral preference factors and fatigue. Specifically, greater ease of getting up in the morning and longer summer outdoor exposure may reduce fatigue risk, while evening chronotype increases susceptibility.

## 1. Introduction

With the rapid development of the social economy, global environmental changes, population aging, and the emergence of novel diseases, fatigue has emerged as a critical public health challenge under the influence of multiple interacting factors. A large-scale global study involving 623,624 participants revealed that 16.4% of the population currently experiences fatigue,^[1]^ with chronic fatigue being particularly detrimental, as it persists despite rest and leads to severe consequences. It reduces work efficiency by up to 75%,^[2]^ and exacerbates underlying diseases, including an increased risk of Hashimoto’s thyroiditis (10-20%) and immune dysfunction in ME/chronic fatigue syndrome patients (CFS) patients (25% showing deficiencies in total immunoglobulins or immunoglobulin subclasses, 15% lacking mannose-binding lectin, and 25% demonstrating polyclonal immunoglobulin proliferation),^[3]^ and is associated with elevates mortality risk (CFS patients show an all-cause standardized mortality ratio of 1.14; 95% CI = 0.65–1.85).^[4]^ Furthermore, it severely diminishes quality of life, leaving one-third of patients bedridden,^[2]^ and imposes substantial economic burdens, with annual costs averaging $20,000 per case.^[5–7]^ Occupational fatigue among specific groups, such as drivers (contributing to 10-40% of fatigue-related traffic accidents), firefighters (10–20% suffering duty-related injuries due to impaired balance) and healthcare workers (who faces a 2.3-fold higher risk of adverse events or medication errors when fatigued)^[8]^ poses severe threats to productivity and public safety, potentially resulting in irreversible loss of life and economic damage.

Fatigue arises from complex interactions among environmental, behavioral, genetic, and disease-related factors, with existing research identifying multiple contributors, including circadian rhythm disruption (manifested through reduced light exposure, irregular activity patterns, delayed melatonin secretion, and disrupted body temperature rhythms),^[9]^ overcrowded spaces, environmental stressors (54.9% of CFS patients report light sensitivity that exacerbates fatigue, while 62.8% experience noise-induced fatigue aggravation),^[10]^ occupational pressures (77.3% of healthcare workers developed burnout during the COVID-19 pandemic),^[11]^ and sleep deprivation (sleep disruption and non-restorative sleep affect 95% of ME/CFS patients, with unstable non-rapid eye movement sleep being linked to fatigue symptoms).^[12]^ Genetic factors, including candidate genes such as *ECE2, GPX1, METTL21EP, RP11-665J16.1*, and *SNF8*, have also been associated with fatigue.^[13]^ While these findings provide a basis for intervention strategies, most existing research relies on cross-sectional surveys, retrospective studies, or qualitative interviews, which are limited by confounding factors and reverse causality. For example, it remains unclear whether cortisol reduction in CFS patients is a cause or consequence of the condition.^[14]^ Similarly, while indoor environmental factors like temperature, humidity, light, and noise are known to exacerbate fatigue in healthcare settings, their relative contributions remain undefined, which hinders precise interventions. These knowledge gaps underscore the need for Mendelian randomization (MR) analysis, an emerging causal inference method that leverages genetic variants as instrumental variables (IVs) to investigate the causal relationships between exposures (e.g., biomarkers, behaviors, or environmental factors) and health outcomes.^[15]^ By mimicking the logic of randomized controlled trials (RCTs), MR addresses challenges inherent in traditional observational studies. This investigation specifically aims to elucidate genetically validated causal relationships between modifiable behavioral preference factors and fatigue susceptibility using genetic variants as instrumental variables.

## 2. Methods

### 2.1. Research design and data sources

**Figure 1.**
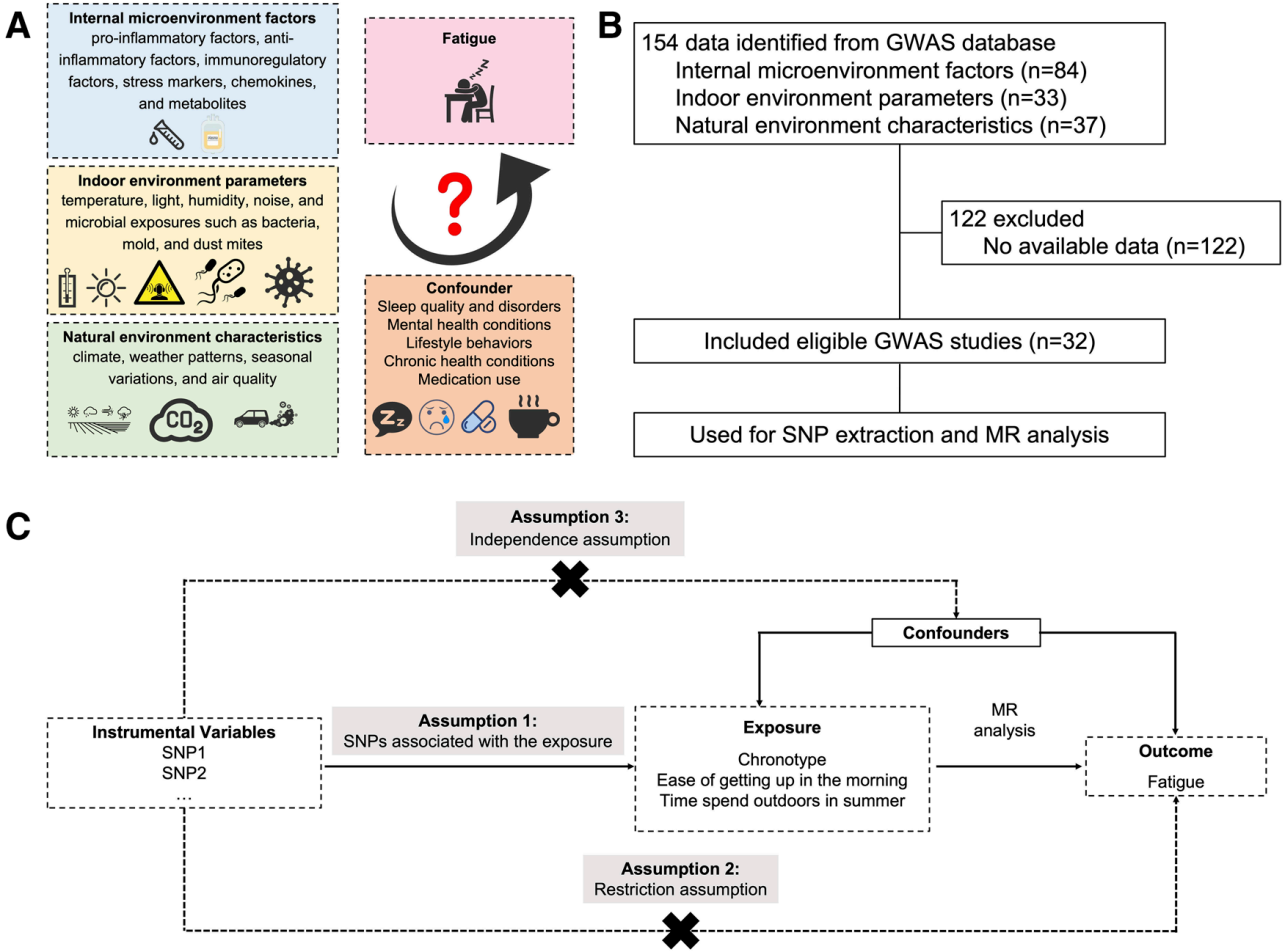
Design of Mendelian randomization study. (A) Potential environmental and confounding factors associated with fatigue. (B) Flowchart of GWAS data selection process. (C) Conceptual framework of MR assumptions.

Our MR analysis utilized GWAS summary statistics from the Neale Lab and MRC-IEU database, encompassing comprehensive environmental and behavioral- related measures: internal microenvironment (including pro-inflammatory factors [TNF-α, IL-1β, IL-6, IL-8], anti-inflammatory factors [IL-4, IL-10, TGFβ], immunoregulatory factors [IL-2, IFN-γ], stress markers [cortisol, C-reaction protein], chemokines, and metabolites [lactate, reactive oxygen species, glucose, ketones]); indoor environment (temperature, light, humidity, noise, and microbial exposures [bacteria, mold, and dust mites]); natural environment (climate zones [tropical, temperate, polar], weather patterns [precipitation, wind speed, UV index, atmospheric pressure], seasonal variations [photoperiod, diurnal temperature variation], and air quality [PM_2.5_, PM_10_, NO_2_, SO_2_, VOCs, CO_2_ concentration]). After screening, a total of 32 GWAS databases were included in our study (Table S2, Supplemental Digital Content 1). Through comprehensive evaluation of potential determinants, our MR study ultimately identified three key exposure-outcome pairs that consistently satisfied the core MR assumptions based on available stastistical tests, although key assumptions such as independence and exclusion restriction cannot be directly verified. These pairs demonstrated: strong genetic instrument-exposure associations (chronotype [n = 301,143; 10,894,596 SNPs], morning alertness [n = 461,658; 9851,867 SNPs], and summer outdoor time [n = 419,314; 9851,867 SNPs]); no evidence of pleiotropic confounding (as validated via MR-Egger intercept tests); and statistically supported exclusive exposure-mediated effects on fatigue outcomes (ICD-10 R53.82; 2076 cases/460,857 controls; 9851,867 SNPs). All GWAS summary statistics were obtained from the IEU OpenGWAS project (https://gwas.mrcieu.ac.uk/, accessed April 13, 2025), with European-ancestry populations and no gender stratification (Table 1), which collectively supports the robustness of our causal inference framework as illustrated in Figure 1.

**Table 1:** Summary of the GWAS included in this MR study.

Factors	Dataset ID	Sample size	SNPs	Population	Consortium	Build
Chronotype	ukb-a-11	301143	10894596	European	Neale Lab	HG19/GRCh37
Ease of getting up in the morning	ukb-b-2772	461658	9851867	European	MRC-IEU	HG19/GRCh37
Time spent outdoors in summer	ukb-b-969	419314	9851867	European	MRC-IEU	HG19/GRCh37
Fatigue	ukb-b-8961	ncase:2076/ncontrol: 460857	9851867	European	MRC-IEU	HG19/GRCh37

Output from GWAS pipeline using Phesant derived variables from UKBiobank.

### 2.2. Instrumental variables

The instrumental variables (IVs) must adhere to three core assumptions as detailed in the research design. All analyses were conducted using R 4.1.2 with the “TwoSample MR” and “MR-PRESSO” packages. Using a genome-wide significance threshold of *P* < 5 × 10^−8^, we selected SNP loci significantly associated with chronotype, ease of getting up in the morning, and time spent outdoors in summer as the IVs for initial screening. We then applied stringent quality controls, including linkage disequilibrium (LD) clumping (r^2^ = 0.001, the region width = 10,000 kb) and exclusion of outcome-associated SNPs (*P* < 5 × 10^−8^ for fatigue). Instrument strength was verified (F-statistic > 10) to minimize weak instrument bias. For missing SNPs in the fatigue GWAS, we employed proxy SNPs (r^2^ > 0.8) where available. Finally, we conducted MR-PRESSO analysis to detect and remove pleiotropic outliers prior to performing causal estimates, thereby ensuring the validity of our genetic instruments. All exposure and outcome datasets were harmonized and summarized separately, with removal of fatigue-associated SNPs to satisfy MR assumptions.

### 2.3. Univariate, multivariate MR and sensitivity analysis

Our MR analysis employed five complementary methods, including inverse variance weighted (IVW; used as the primary method due to superior statistical efficiency), MR-Egger, weighted median estimator (WME), weighted mode, and simple mode. MVMR was additionally performed to assess the independence of exposure effects on fatigue outcomes. To ensure robustness, we conducted several sensitivity analyses: Cochran’s Q test (where *P* > .05 indicates no significant evidence of heterogeneity); MR-Egger regression (where an intercept *P* < .05 would suggest horizontal pleiotropy); and Leave-one-out validation, in which sequential exclusion of individual instruments was performed to assess whether effect estimates remained consistent. Collectively, this integrative analytical framework coupled with rigorous sensitivity testing supports the robustness of our causal findings, while acknowledging the inherent limitations of MR analyses.

## 3. Results

### 3.1. Selection of instrumental variables

We systematically selected IVs through a multi-step quality control process. Initial screening identified 61 SNPs associated with chronotype, 61 with morning alertness, and 39 with summer outdoor time, all significantly associated with their respective exposures (*P* < 5 × 10^−8^). Of these, we excluded 2, 2, and 3 SNPs respectively owing to their association with the outcome. Notably, we identified several palindromic SNPs with intermediate allele frequencies across exposures: rs4417706 and rs4729854 for chronotype, rs11629621 and rs1914397 for morning alertness, and rs188581, rs28837979, and rs6906737 for outdoor time. After implementing MR-PRESSO analysis (which detected no outliers) and completing quality control procedures, our final set comprised 59 chronotype SNPs, 59 morning alertness SNPs, and 36 outdoor time SNPs. These instruments collectively explained 2.5%, 1.6%, and 2.3% of the variance in their respective exposures (R^2^). Importantly, all retained SNPs demonstrated robust instrument strength, with F-statistics ranging as follows: 25.65–74.49 for chronotype, 17.67–75.12 for morning alertness, and 23.14–95.26 for outdoor time, effectively eliminating concerns about weak instrument bias. Comprehensive SNP characteristics are presented in Table 2 and Table S1, Supplemental Digital Content 2.

**Table 2:** The SNPs of chronotype, ease of getting up in the morning, and time spent outdoors in summer associated with fatigue.

SNP	EA	OA	EAF	CHR	POS	β	SE	*P*	R^2^	F
**Chronotype-61**										
rs10189912	G	A	0.369	2	144162609	<-0.0001	<0.001	.700	<0.001	47.088
rs10280205	C	T	0.309	7	24082063	<0.0001	<0.001	.850	<0.001	28.703
rs10495976	T	A	0.390	2	49750698	<-0.0001	<0.001	.800	<0.001	28.916
rs10520176	C	T	0.500	2	77217310	<-0.0001	<0.001	.420	<0.001	64.485
rs1067404	G	T	0.757	2	44675823	<-0.0001	<0.001	.580	<0.001	43.538
……										
**Ease of getting up in the morning-61**										
rs1017168	C	A	0.644	12	107435405	<-0.0001	<0.001	.036	<0.001	23.320
rs10175975	T	C	0.182	2	59429807	<-0.0001	<0.001	1.000	<0.001	20.733
rs10455248	T	C	0.282	6	72482458	<0.0001	<0.001	.940	<0.001	18.405
rs10462020	G	T	0.196	1	7880683	<0.0001	<0.001	.380	<0.001	39.758
rs10518446	C	G	0.177	1	77775233	<-0.0001	<0.001	.084	<0.001	48.773
……										
**Time spent outdoors in summer-39**										
rs1028455	A	T	0.329	14	88829975	<-0.0001	<0.001	.180	<0.001	26.007
rs10984444	C	A	0.475	9	121980347	<0.0001	<0.001	.460	<0.001	28.930
rs11776021	A	G	0.228	8	93025183	<-0.0001	<0.001	.910	<0.001	31.460
rs117799466	C	G	0.337	15	34659517	<-0.0001	<0.001	.700	<0.001	27.144
rs12203592	T	C	0.220	6	396321	<-0.0001	<0.001	.300	<0.001	29.248
……										

Note: More SNPs information can be found in Table S1, Supplemental Digital Content 2.

### 3.2. Univariate and Multivariate MR analysis

**Figure 2.**
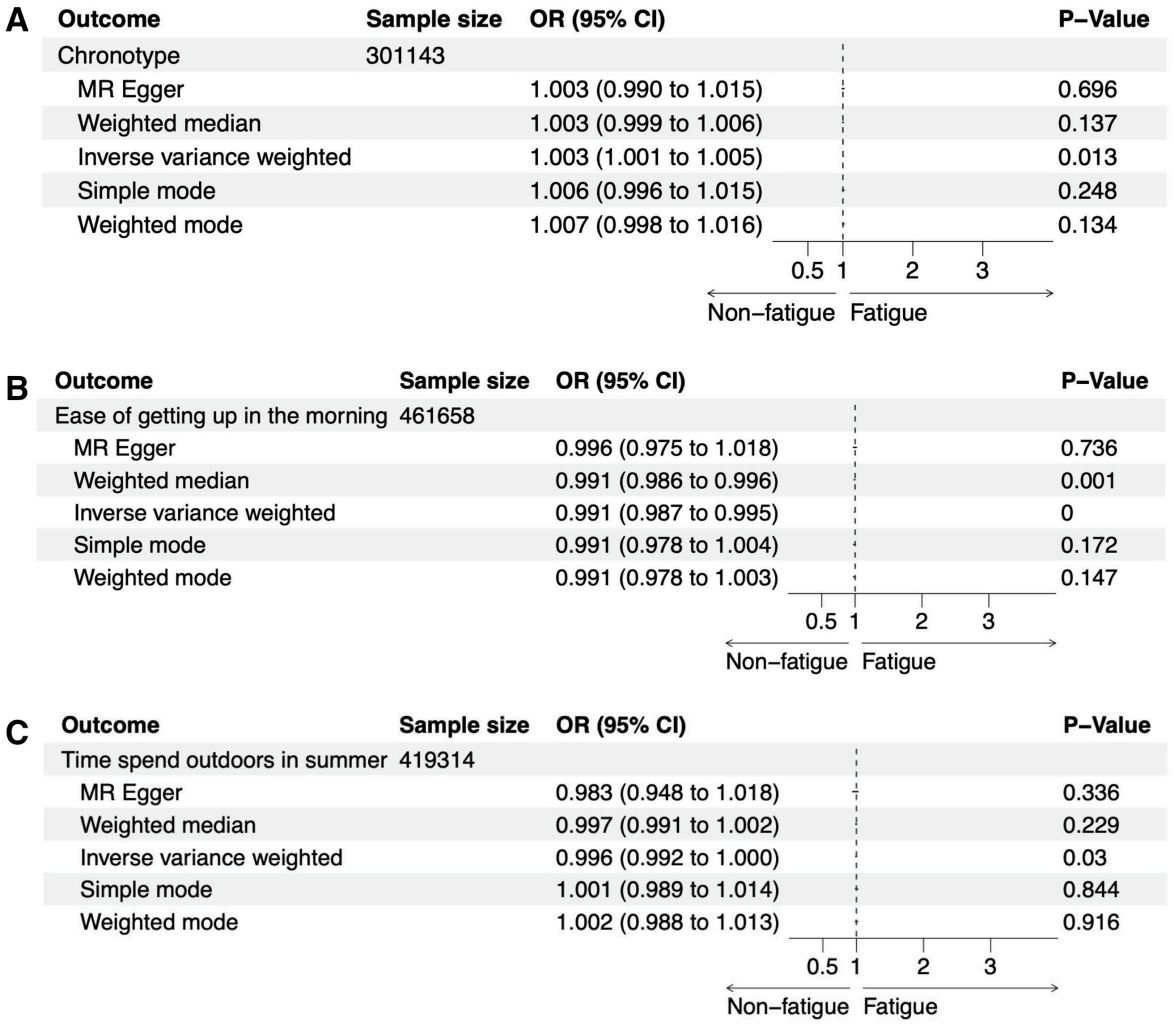
Forest plot of MR analysis. (A) the relationship between chronotype and fatigue, (B) the relationship between ease of getting up in the morning and fatigue, (C) the relationship between time spending outdoors in summer and fatigue.

**Figure 3.**
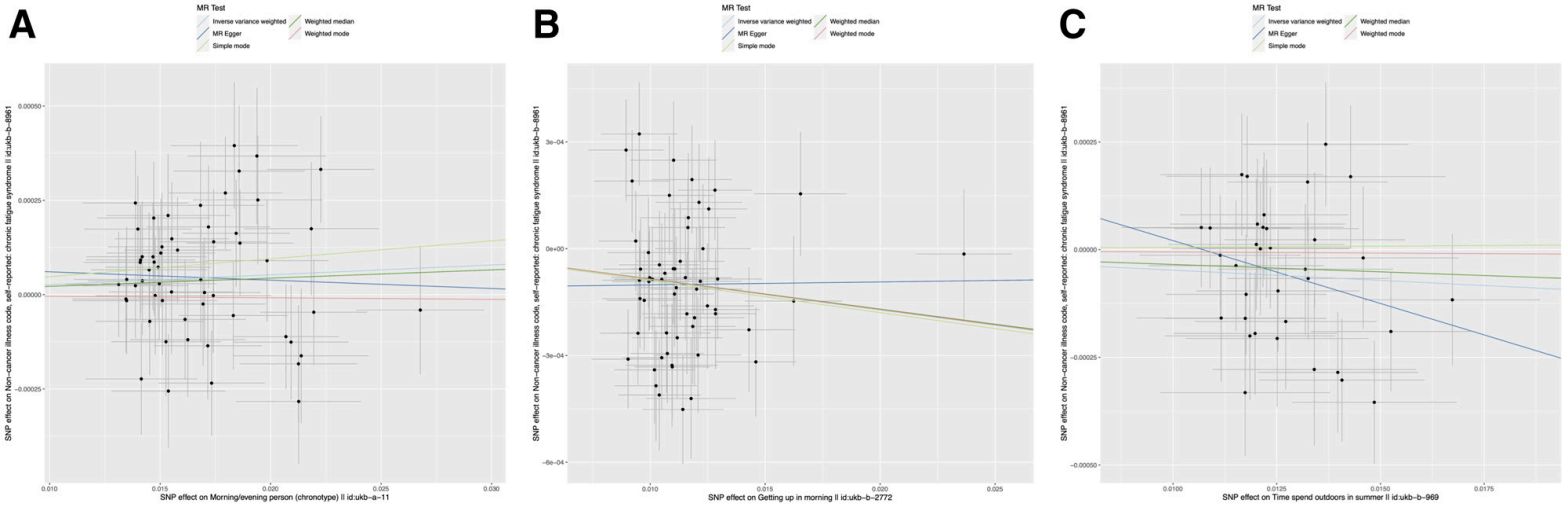
Scatter plot of MR analysis. (A) chronotype on fatigue, (B) ease of getting up in the morning on fatigue, (C) time spending outdoors in summer on fatigue.

Genetically predicted lower ease of getting up in the morning and shorter summer outdoor exposure duration were associated with an increased risk of fatigue, while a genetically predicted greater tendency toward evening chronotype was associated with an increased risk of fatigue (Table 3, Figures 2 and 3, and Figure S1, Supplemental Digital Content 3). The IVW analysis demonstrated statistically significant effects: chronotype (OR = 1.003, 95%CI: 1.001~1.005, *P* = .013), ease of getting up in the morning (OR = 0.991, 95%CI: 0.987~0.995, *P* < .001), and summer outdoor exposure duration (OR = 0.996, 95%CI: 0.992~1.000, *P* = .030). After adjusting for the effects of all three indicators, MVMR analysis showed that greater ease of getting up in the morning remained significantly associated with a lower risk of fatigue (OR = 0.987, 95%CI: 0.980~0.995, *P* = .002), as shown in Table 4.

**Table 3:** Univariate MR results of the relationship among chronotype, ease of getting up in the morning, time spent outdoors in summer and fatigue.

Methods	Sample size	OR	95%CI	*P*
**Chronotype on Fatigue**	301143			
MR Egger		1.003	0.990-1.015	.696
Weighted median		1.003	0.999-1.006	.137
Inverse variance weighted		1.003	1.001-1.005	.013
Simple mode		1.006	0.996-1.015	.248
Weighted mode		1.007	0.998-1.016	.134
**Ease of getting up in the morning on Fatigue**	461658			
MR Egger		0.996	0.975-1.018	.736
Weighted median		0.991	0.986-0.996	.001
Inverse variance weighted		0.991	0.987-0.995	<.001
Simple mode		0.991	0.978-1.004	.172
Weighted mode		0.991	0.978-1.003	.147
**Time spent outdoors in summer on Fatigue**	419314			
MR Egger		0.983	0.948-1.018	.336
Weighted median		0.997	0.991-1.002	.229
Inverse variance weighted		0.996	0.992-1.000	.030
Simple mode		1.001	0.989-1.014	.844
Weighted mode		1.002	0.988-1.013	.916

**Table 4:** Results of multivariate Mendelian randomization studies.

MVMR	Characteristic	SNPs	β	SE	OR (95%CI)	*P*
Ease of getting up in the morning on fatigue	Chronotype	46	−0.004	0.003	0.996 (0.991–1.001)	.081
	Ease of getting up in the morning	44	−0.013	0.004	0.987 (0.980–0.995)	.002
	Time spent outdoors in summer	29	−0.004	0.002	0.996 (0.992–1.000)	.065

### 3.3. Sensitivity analysis

**Figure 4.**
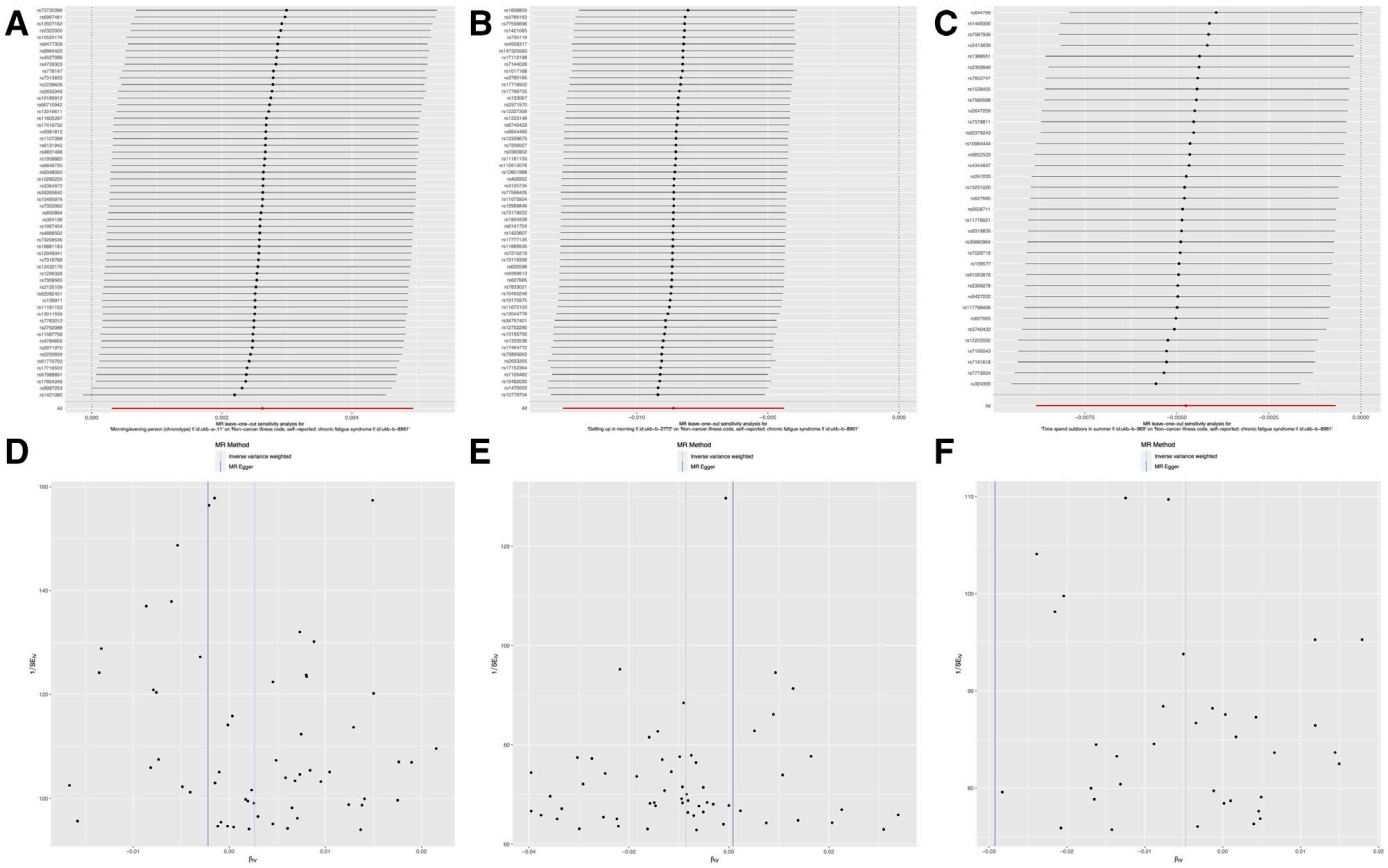
Sensitivity analysis of MR analysis. (A–C) Results of leave-one-out sensitivity analysis; (D–F) Funnel plot of MR analysis. (A,D) chronotype on fatigue, (B,E) ease of getting up in the morning on fatigue, (C,F) time spending outdoors in summer on fatigue.

For the association between chronotype and fatigue, Cochran’s Q statistics from both the IVW and MR-Egger methods indicated no evidence of heterogeneity (IVW: Q = 59.950, *P* = .405; MR-Egger: Q = 59.946, *P* = .369). Similarly, no significant heterogeneity was observed in the analysis of time spent outdoors in summer and fatigue (IVW: Q = 39.367, *P* = .281; MR-Egger: Q = 38.750, *P* = .264). In contrast, evidence of heterogeneity was detected in the analysis of ease of getting up in the morning and fatigue (IVW: Q = 85.581, *P* = .016; MR-Egger: Q = 82.242, *P* = .013). No evidence of horizontal pleiotropy was observed across all analyses, as indicated by non-significant MR-Egger intercepts (all *P* > .05). In addition, MR-PRESSO analysis did not identify any outlier SNPs (Table 5). Additional sensitivity analyses further supported the potential associations. Leave-one-out analysis showed that no single SNP disproportionately influenced the causal estimates (Fig. 4), and funnel plots displayed symmetrical distributions of SNP effects (Fig. 4), suggesting no strong evidence of directional bias, despite heterogeneity observed for ease of getting up in the morning. Overall, although heterogeneity was observed for ease of getting up in the morning, the consistency across multiple MR methods and sensitivity analyses indicates that the main findings are unlikely to be strongly biased. These results suggest potential associations between behavioral preference-related traits and fatigue risk, although the presence of significant heterogeneity for ease of getting up in the morning warrants more cautious interpretation of the robustness of this specific finding.

**Table 5:** Sensitivity analysis of MR study.

Exposure	Outcome	Heterogeneity test				Pleiotropy test			MR-PRESSO test	
		IVW		MR-Egger		MR-Egger			Outlier	*P*
		Q	*P*	Q	*P*	Intercept	SE	*P*		
Chronotype	Fatigue	59.950	.405	59.946	.369	<0.001	<0.001	.950	NA	.288
Ease of getting up in the morning	Fatigue	85.581	.016	82.242	.013	<-0.001	<0.001	.632	NA	.023
Time spent outdoors in summer	Fatigue	39.367	.281	38.750	.264	<0.001	<0.001	.467	NA	.179

## 4. Discussion

Fatigue, characterized by transient decline in physical and cognitive performance, represents a complex physiological state arising from excessive exertion, inadequate recovery, and psychological stress.^[16]^ While observational studies have reported associations between environmental factors and fatigue, the causal nature of these relationships remains uncertain. Using MR analysis, a method that leverages genetic variants as IVs to minimize confounding, we provide evidence suggesting potential causal effects of chronotype, ease of getting up in the morning, and summer outdoor exposure duration on fatigue risk. Specifically, a genetic predisposition toward greater morning alertness was associated with reduced fatigue risk, whereas lower morning alertness was linked to heightened fatigue risk. Crucially, our findings indicate that greater ease of getting up in the morning acts as a protective factor against fatigue; therefore, mechanistic interpretations should focus on this protective direction. Genetic variants related to morning functioning may modulate cortisol regulation and stress-response pathways. Difficulty in getting up in the morning, rather than early waking itself, may reflect underlying circadian misalignment or poor restorative sleep, both of which are recognized contributors to fatigue. Moreover, difficulty in getting up in the morning may partly share genetic overlap with mood disorders such as depression and anxiety, both of which are closely linked to fatigue. As such, reduced ease of getting up in the morning may act as a proxy for underlying neuropsychiatric traits that confer increased fatigue susceptibility. Environmental factors, including social jetlag, where individuals with misaligned sleep-wake preferences are forced to adapt to late-night societal schedules, can exacerbate this risk. Shortened sleep duration and fragmented sleep architecture likely contribute to accumulated fatigue, particularly when biological and social rhythms fall out of sync. Taken together, these findings highlight the intricate interplay between circadian biology, mental health, and environmental contexts in shaping fatigue risk, calling for further investigation into the underlying mechanisms and targeted interventions.

Chronotype, defined as an individual’s circadian preference for sleep-wake patterns and daily activity rhythms, significantly influences fatigue susceptibility. Standard chronotype assessments include actigraphy-measured sleep midpoint (aMS) and the Morningness-Eveningness Questionnaire (MEQ), which categorize individuals into morning, intermediate, and evening types.^[17]^ Consistent with previous observational studies,^[17–19]^ our genetic analysis suggests that evening chronotype is associated with increased fatigue risk. The mechanisms by which chronotype influences fatigue remain not yet fully understood but may invovle genetic polymorphisms in circadian clock genes (e.g., *PER3, CLOCK*), dysregulation of cortisol and other hormonal rhythms, misalignment between internal circadian timing and external schedules, as well as disrupted sleep architecture with reduced sleep quality. These findings highlight the importance of considering chronobiological factors in fatigue management, while underscoring the need for further research to elucidate the molecular pathways linking chronotype to fatigue, investigate chronotype-specific interventions, and examine gene-environment interactions in fatigue development.

Furthermore, shorter summer outdoor exposure duration was associated with a higher risk of fatigue, whereas longer summer outdoor exposure duration appeared protective in our MR analysis. However, the relationship between outdoor exposure and fatigue may be context-dependent, particularly influenced by environmental conditions such as temperature and activity intensity. Previous observational studies have suggested that under certain conditions, especially extreme heat, outdoor exposure may instead exacerbate fatigue. A Canadian cohort study reported that high outdoor temperatures constitute a contributing factor to fatigue occurrence, with the risk increasing as temperatures rise.^[20]^ Similarly, an investigation using National Collegiate Athletic Association (NCAA) athlete data revealed significant performance declines (20-40%) under critical heat stress, with running distances decreasing markedly when wet-bulb globe temperature (WBGT) exceeded specific thresholds relative to humidity levels: > 28°C at RH < 50%, > 25°C at RH 50–75%, or > 23°C at RH > 75%. These impairments were particularly pronounced during later competition stages, attributable to cumulative heat- and humidity-induced fatigue mediated by thermoregulatory overload, cardiovascular strain, and metabolic disruption.^[21,22]^ Importantly, the protective effect observed in our MR analysis likely reflects genetically predicted habitual outdoor behavior under typical environmental conditions, rather than acute exposure to extreme heat or high-intensity physical stress. Thus, the apparent discrepancy between MR findings and observational or athletic studies may be explained by differences in exposure context, duration, and intensity. Mechanistically, heat-induced fatigue arises from interconnected physiological disruptions including thermoregulatory imbalance, metabolic dysfunction, oxidative stress cascades, and inflammatory activation.^[23–26]^ Taken together, these findings suggest that moderate and regular outdoor exposure may be beneficial, whereas extreme environmental conditions may offset or even reverse this effect.

Collectively, the MR investigation suggests potential associations between key behavioral preference-related factors and fatigue susceptibility. These findings substantially advance our understanding of behavioral and circadian determinants in health by: offering genetically validated evidence for modifiable risk factors, and pinpointing specific intervention targets related to circadian alignment, enhancement of morning functioning, and optimization of outdoor behavioral patterns. The results offer a preliminary evidence-based framework for devising precision prevention strategies in occupational, clinical, and public health settings.

While our Mendelian randomization study suggests potential causal relationships, several limitations warrant consideration. First, despite using large GWAS datasets (N = 1645,048), the fatigue outcome comprised relatively few cases (n = 2076), which may constrain statistical power for small effect sizes. Second, although we rigorously excluded pleiotropic SNPs, residual horizontal pleiotropy may persist due to unmeasured biological pathways. Third, all data were derived from European-ancestry populations, which limits generalizability to other ethnic groups, a critical gap given known circadian rhythm variations across ancestries. Fourth, the GWAS exposure measures (e.g., self-reported chronotype) may not capture subtle environmental exposures with comparable precision. Notwithstanding these limitations, our research findings provide preliminary evidence supporting the contribution of behavioral and circadian factors to fatigue pathogenesis.

## 5. Conclusion

To sum up, this MR study suggests potential genetic evidence supporting associations between modifiable behavioral preference factors, namely chronotype, morning alertness, and summer outdoor exposure duration, and fatigue risk. More specifically, evening chronotype was linked to increased fatigue risk, whereas greater ease of getting up in the morning and longer summer outdoor exposure duration were associated with reduced fatigue risk. Of note, greater ease of getting up in the morning emerged as a potential independent protective factor against fatigue. Nevertheless, the limited number of fatigue cases in the outcome GWAS, along with the inherent limitations of MR, should be considered when interpreting these findings. Further research is warrented to corroborate our conclusions and elucidate the underlying biological mechanisms and potential intervention strategies.

## Acknowledgements

We are grateful to the researchers who provided the original data.

## Author contributions

**Data curation:** Tao Luo, Fen Zhang, Hui Xue, Lanqing Wang.

**Methodology:** Tao Luo, Hui Xue, Lanqing Wang.

**Writing – original draft:** Tao Luo, Fen Zhang, Lanqing Wang.

**Investigation:** Fen Zhang, Lanqing Wang.

**Writing – review & editing:** Fen Zhang, Hui Xue, Lanqing Wang.

**Funding acquisition:** Hui Xue.

**Conceptualization:** Lanqing Wang.






